# Relationship among Dietary Intake of Vitamin E, Lipid Peroxidation Markers, and C-Reactive Protein in Flu-Like Patients Diagnosed with COVID-19

**DOI:** 10.1155/2023/8889213

**Published:** 2023-11-06

**Authors:** Maísa Guimarães Silva Primo, Liriane Andressa Alves da Silva, Vanessa Brito Lira de Carvalho, Margarete Almeida Freitas de Azevedo, Nayara Vieira do Nascimento Monteiro, Vitória Ribeiro Mendes, Jaynara Keylla Moreira da Silva, Amanda Suellenn da Silva Santos Oliveira, Ana Karolinne da Silva Brito, Ana Lina de Carvalho Cunha Sales, Jacenir Reis dos Santos Mallet, José Miguel Luz Parente, Emídio Marques de Matos Neto, Paulo Michel Pinheiro Ferreira, Daniel Dias Rufino Arcanjo, Maria do Carmo de Carvalho e Martins

**Affiliations:** ^1^Department of Nutrition, Federal University of Piauí, Teresina, Piauí, Brazil; ^2^Department of Biophysics and Physiology, Federal University of Piauí, Teresina, Piauí, Brazil; ^3^University Hospital of Piauí, Federal University of Piauí, Teresina, Piauí, Brazil; ^4^Oswaldo Cruz Institute (Fiocruz), Rio de Janeiro, Brazil; ^5^Department of Physical Education, Federal University of Piauí, Teresina, Piauí, Brazil

## Abstract

**Objective:**

This research aimed to assess the intake of vitamin E and its relationship with lipid peroxidation markers and C-reactive protein levels in patients with flu symptoms and COVID-19 diagnosis.

**Methods:**

A cross-sectional study with 121 patients of both sexes assisted at two basic health units in the city of Teresina, Piauí, with COVID-19 diagnosis confirmed through real-time reverse transcription polymerase chain reaction, was performed between the 3^rd^ and 7^th^ days of flu symptoms. The global nutritional status and the measurement of waist circumference were assessed according to the World Health Organization recommendations. The dietary energy intake, macronutrients, and vitamin E consumption were assessed through the 24 hr food recall method. The malondialdehyde plasmatic concentration (MDA) was measured through the method of thiobarbituric acid-reactive substances. Myeloperoxidase (MPO) was assessed through the oxidation speed of the o-dianisidine substrate in the presence of hydrogen peroxide. C-reactive protein (CRP) levels were measured by a high-sensitivity immunoturbidimetry method.

**Results:**

The most common symptoms reported by the participants were sore throat, fever, and cough. Regarding the global nutritional status evaluation, the majority of the sample had overweight. The dietary intake of vitamin E was 100% inadequate and presented a mild correlation (*r* = 0.197) with MDA, a redox status marker. No correlation was observed among MPO, CRP, and the dietary intake of vitamin E.

**Conclusion:**

The dietary intake of vitamin E was related to MDA as the marker of redox status.

## 1. Introduction

COVID-19, a contagious disease caused by the new coronavirus (SARS-CoV-2), is transmitted by respiratory droplets or contact with contaminated surfaces and its incubation period varies from 1 to 14 days [1, 2]. The clinical manifestations of COVID-19 vary from mild infections of the upper respiratory tract to severe pneumonia, leading to a syndrome of serious acute respiratory discomfort. There are also accounts of symptoms such as headache, dyspnea, nausea, vomiting, diarrhea, loss of appetite, smell, and taste [3].

In addition, the new coronavirus activates the nicotinamide adenine dinucleotide phosphate (NADPH) oxidase, causing an increase in reactive oxygen species (ROS) such as superoxide anion and hydrogen peroxide, what may result in oxidative stress and contribute to the inflammatory responses that are activated by transcriptions factors, like NF-kb, and exacerbated by the nucleotide-binding domain and leucine-rich repeat-containing family pyrin domain-containing 3 inflammasome [4, 5].

Up to the present moment, there has been no effective treatment for the infection by the new coronavirus [6]. Strengthening the immune systems by means of adequate nutritional status, along with a balanced diet, is likely to be essential in the fight against the virus, due to the modulation of the inflammatory process and the decrease in the occurrence of oxidative stress [7]. Among the nutrients with antioxidant and anti-inflammatory properties that may aid in this process is vitamin E [8, 9].

This vitamin is present in the cell membrane, mainly in the cells of the immune system, with action in the differentiation of the immature T-cells in the thymus [10]. The recommended intake of vitamin E directed to healthy adults, considering the recommended dietary allowance for vitamin E (in the form of *α*-tocopherol) is 15 mg/day for women and adult men. [11].

Considering that lipid peroxidation is able to damage the membranes of the immune cells, vitamin E contributes to the protection of those cellular membranes against oxidative attacks and fluidity maintenance, integrity and functionality of the immunological cells [12]. Despite a possible adjuvant action of vitamin E to prevent the risk of respiratory diseases such as COVID-19 through its immunomodulating and antioxidant functions, there are few studies on the intake of this vitamin among people infected with the new coronavirus. Hence, the present work assessed the vitamin E intake and its relationship with lipid peroxidation and C-reactive protein in patients with flu symptoms and COVID-19 diagnosis.

## 2. Methods

### 2.1. Type of Study, Setting, and Sample

Cross-sectional study, which consists of a subproject of a macro project titled: “randomized clinical study of the effects of atazanavir, daclatasvir, and ivermectin in ambulatory patients with a positive result for COVID-19,” performed in basic health units (BHUs) for the assistance to patients with symptoms of flu syndrome, with suspicion and/or confirmed diagnosis of COVID-19, in the city of Teresina, capital of the state of Piauí, Brazil. The research was conducted in adult participants, of both sexes, who had had symptoms of flu syndrome and a further diagnosis of COVID-19, undergoing ambulatory treatment in the BHUs.

The sample was determined by a nonprobabilistic methodology, conducted in patients who spontaneously attended two BHUs located in two different neighborhoods of the city of Teresina: East Zone (Planalto Uruguai) and South Zone (Parque Piauí), from November/2020 to March/2021 (120 days). The choice of the BHUs was based on the fact that they are the ones with the highest amount of assistance among the 19 BHUs providing care for flu syndrome with suspicion or confirmed diagnosis of COVID-19.

### 2.2. Eligibility Criteria

The study received patients aged 18 or older, of both sexes, with a diagnosis of COVID-19 confirmed through real-time reverse transcription polymerase chain reaction (RT-PCR) made between the 3^rd^ and 7^th^ days of symptoms of flu syndrome (fever, dry cough, dyspnea, thoracic pain, headache, loss of smell, and taste) in ambulatory treatment.

Exclusion criteria consisted of participants recommended with hospitalization in the first medical appointment, pregnant and lactating women, as well as those patients with a previous diagnosis of H1N1, AIDS/HIV, epilepsy, chronic or acute pancreatitis, cancer, and hepatic and renal problems, and those making use of nutritional supplements.

### 2.3. Recruiting and Data Collection

Patients who met the eligibility criteria and, upon orientations, consent to participate in the study responded to a social-economic questionnaire with clinical information. The participants also responded to a 24 hr recall questionnaire. The anthropometric measures of weight, height, and waist circumference (WC) were gauged. The research comprised patients with a COVID-19 diagnosis confirmed by means of positive RT-PCR testing and who did not receive any medication such as azithromycin, atazanavir, daclatasvir, and ivermectin.

### 2.4. Testing for the COVID-19 Diagnosis

Samples of nasopharynx swab for RT-PCR were collected in 15 mL Falcon tubes in viral transport media by a qualified professional in the BHUs following the biosecurity recommendations.

The detection of the SARS-CoV2 virus RNA was made with the RT-PCR technique using the *StepOne plus* equipment (*Applied Biosystems*). The amplification protocols were defined according to the kit. The results were expressed as “detectable” or “not detectable.” The exams were made at the “Dr. Costa Alvarenga” Central Laboratory of Public Health (LACEN-PI).

### 2.5. Anthropometric Assessment

The anthropometric assessment was made by gauging weight (kg) and height (m), used for the assessment of the body mass index (BMI) and the classification of the global nutritional status was calculated according to cutting scores proposed for adults by the World Health Organization [13].

WC (cm) was measured with a flexible and nonextensible measuring tape, positioned surrounding the natural line of the waist, on the average point between the last rib and the iliac crest [14]. The classification of the values was performed according to the reference proposed for the Brazilian population, of both sexes, by the WHO [15].

### 2.6. Evaluation of the Dietary Intake

Dietary intake was estimated through the 24 hr recall method applied on two nonconsecutive days during the diagnosis. The patients were interviewed regarding food, beverages, and their amounts consumed over the 24 hr immediately before the interview, including the brands of the industrialized foods. Photograph albums with pictures of foods and servings were shown to assist in a more accurate measuring of the amounts consumed [16].

The data obtained were analyzed regarding the energy and macronutrient contents with the *Dietpro*® software 6.0 (2019). Adequation of the diet regarding macronutrients was assessed considering the recommendations proposed by the Institute of Medicine [11], as established in the acceptable macronutrient distribution range (AMDR) and the vitamin E intake by the DRIs.

In order to diminish errors of unexplained variability, such as intraindividual and interindividual, the multiple source method proposed by Haubrock et al. [17] and Harttig et al. [18] was applied, which uses a model of multiple regression in function of covariables such as sex and age.

### 2.7. Assessment of the Lipid Peroxidation Biomarkers

#### 2.7.1. Determination of the Malondialdehyde Plasmatic Concentration

The malondialdehyde (MDA) concentrations were determined by the production of thiobarbituric acid-reactive species, as described by Ohkawa et al. [19] with adaptations. In short, 200 *µ*L of plasma was added to tubes containing acetic acid pH 3.5 and thiobarbituric acid 0.5%, heated in a water bath at 85°C for 60 min. 50 *µ*L of 8.1% sodium dodecyl sulfate was added after a 15 min ice bath. The tubes were centrifuged for 15 min at 12,000 rpm at 25°C. The reading was performed on a spectrophotometer in the wavelengths of 532, 510, and 560 nm for the calculation of absorbance corrected to minimize the interference of both the heme pigments and hemoglobin [20]. The results were expressed in nmol of MDA per mL of plasma.

#### 2.7.2. Determination of the Activity of Myeloperoxidase in the Plasma

The myeloperoxidase (MPO) plasmatic concentrations were determined based on the speed of oxidation of the o-dianisidine substrate in the presence of hydrogen peroxide (H_2_O_2_) and verified according to the change in absorbance [21]. A mixture was prepared with 10 *µ*L of plasma and 200 *µ*L of reading solution composed by distilled water, phosphate buffer (pH 6.0), hydrogen peroxide, and o-dianisidine. The speed of oxidation of o-dianisidine was controlled by following the increase in absorbance of the mixture after 1 min on a plate reader with 450 nm. The results were expressed as units of MPO per microliter of plasma (U/*µ*L).

### 2.8. Assessment of the Serum Concentration of C-Reactive Protein

The evaluation of the C-reactive protein (CRP) concentration was performed by the high-sensitivity immunoturbidimetry method, using ELISA kit (Labtest®). The analysis was made according to the manufacturer's recommendations. Values > 5 mg/L were considered as indicators of the presence of inflammatory processes.

### 2.9. Data Analysis

Initially, the normality of the quantitative data distribution was assessed through Shapiro–Wilk test. The chi-square test was performed for categorical variables of cardiometabolic risk represented by WC. In order to test the relations among numerical variables with normal data distribution, Pearson's linear test of correlation was applied; for non-normal distribution, Spearman's test was applied. For all tests, *p*  < 0.05 was considered with a 95% confidence interval. The SPSS software for Windows®, version 22.0 (2013), was used for statistical analysis.

## 3. Results

The sample was composed by 121 patients with positive diagnosis for COVID-19. Regarding the social-economical characteristics of the patients included in this study, there was a predominance of males (54.5%), mean age of 34.22 ± 10.4 years old. Almost half the participants reported a monthly family income of one minimum monthly wage (42.1%) and 52.9% informed completed high school as educational status. Regular practice of physical activity was informed by 41.3% of the participants (Table 1).

Regarding the clinical characteristics (Table 2), most of the study participants did not show noncommunicable diseases (80.2%), nor did they make use of continuous medication (81.8%). The duration of the symptoms until an appointment at the BHU and the application of the RT-PCR test was between 3 and 5 days for 80.9% of the patients—3 days was more frequent (28.9%). The most common symptoms reported were headache (68.6%), fever (62.8%), cough (61.2%), and body pain (62%).

Table 3 shows the mean values and the standard deviations of anthropometric variables of the patients with COVID-19. Average body weight was 75.3 ± 17.53 kg and BMI of 26.91 ± 5.88 kg/m^2^. Average BMI matched the presence of overweight (Table 3).

The average for WC was 89.22 ± 14.78 cm. When stratified by sex, males presented high and very high risk of 24.4% and 19.7%, respectively, and females presented 20% of high risk and 45.5% of very high risk, revealing a statistically significant difference when sex was compared (*p*  < 0.05) (Table 3).

Table 4 presents the dietary intake regarding energy, macronutrients, and vitamin E. The average energy intake of the study participants was 1,365.22 ± 315.85 kcal/day. For the macronutrients, the carbohydrates presented 77.6% of adequacy, proteins 100%, and lipids 96.7%. The average intake of vitamin E was 3.25 ± 1.64 mg/day.

Table 5 shows the mean values of MDA and MPO in patients with COVID-19.  Table 6 shows the correlation analyses amongst numerical variables regarding the dietary intake of vitamin E, lipids, saturated, monounsaturated and polyunsaturated fats, lipid peroxidation markers, and CRP.

There was a moderate statistical correlation (*p* ≤ 0.001; *r* = 0.483) between the vitamin E intake and the monounsaturated fat intake, as well as a weak correlation between the total intake of lipids, polyunsaturated and saturated fats, and MDA (Table 6). In addition, upon the analysis of BMI, MPO, and the dietary intake of vitamin E (Table 6), there was no statistically significant correlation (*p* > 0.05).

## 4. Discussion

This study was the first to assess the intake of vitamin E and its correlation with the lipid peroxidation biomarkers in patients with mild symptoms of flu syndrome and with a positive diagnosis for COVID-19. The intake of this vitamin was inadequate in the analyzed sample. There was no relationship between the insufficient intake of vitamin E, MPO, and BMI. The intake of vitamin E presented a moderate positive correlation with the intake of monounsaturated fats and a weak correlation with the intake of saturated, polyunsaturated fats, and MDA.

The frequency of overweight was observed in this study and opposing to that, Filippo et al. [22] observed that out of the 213 patients, 26.8% (58) were patients that had been treated in an ambulatory level and that had healthy weight as the diagnostic nutritional status.

Nutritional status seems to be a key factor for the diagnosis of COVID-19, since obesity is related to low-grade inflammation, with a decrease in immune response, increases the risk of serious acute respiratory syndrome (SARS) and potential increase in the expression of the angiotensin 2 converting enzyme (ACE2), an enzyme that participates in the process of entrance of the virus into the organism. These alterations partially account for the relationship between the excess of adiposity and the onset of more serious forms of the disease [23–25].

Regarding the energy intake and macronutrients, macronutrients were in accordance with the AMDR recommendations. This result may be considered positive for patients with viral infections, such as COVID-19, since a balanced and diverse diet with protein rich foods, may contribute to the increase in the resistance to infections with the production of antibodies, in addition to contributing to an adequate nutritional status by helping to modulate the immune system, and oxidative stress and inflammation pathways [26].

The assessment of the dietary intake of this study's participants, according to the values defined by the DRIs, showed a great probability of inadequacy in vitamin E intake. The dietary intake of vitamin E had a moderate correlation with the intake of polyunsaturated fats. It is highlighted that the intake of vitamin E is linked to the intake of lipids. Hence, a higher amount of polyunsaturated fats in the diet needs also a higher amount of this vitamin, since it increases the risk of oxidation due to the number of unsaturations of these fatty acids [27].

A weak correlation was found between the dietary intake of vitamin E and the MDA lipid peroxidation marker. This insufficient ingestion values of vitamin E may contribute to a greater lipid peroxidation, a condition that has the trend of being increased in viral infections such as COVID-19.

A reason for the relation between the low intake of Vitamin E and higher lipid peroxidation is based on the fact that this vitamin has the role of maintaining the fluidity of the membranes due to the participation in the modulation of the oxidative pathways of the eicosanoids and of the prostaglandin synthesis, being able to neutralize ROS and nitrogen, as well as aiding in the immune response mediated by the T-cells [28].

As for MPO, the other peroxidation marker used in this study, there was no relationship with MPO levels. Among the possible reasons for missing the correlation with vitamin E intake, it should be noted that MPO is not directly involved in lipid peroxidation; this enzyme participates in this process because it is a peroxidase that is stored in neutrophils, and these cells, when activated, overproduce NADPH oxidase, resulting in increased production of superoxide anion and conversion into hydrogen peroxide [29]. Besides MPO not being a specific marker, the fact that the average intake of vitamin E in the study group was much lower than the daily recommendation of 15 mg/day for adult men and women could also be a reason for the absence of correlation.

Literature is still scarce regarding the studies on the lipid peroxidation biomarkers in patients with COVID-19, especially in an ambulatory level and due to that, no research was found that could compare such parameters with the present study.

Although the mechanisms involving the pathogenesis of COVID-19 and the oxidative stress are not totally clear, one of the possible mechanisms involved is related to the increase in NADPH oxidase. That happens because when the SARS-CoV-2 virus decreases the availability of ACE2, angiotensin II (Ang II) becomes available to interact with AT1R (receptor type 1 of Ang II), stimulating the activation of NADPH oxidase and inducing the increased production of ROS, including hydrogen superoxide and peroxide, as well as exacerbated anti-inflammatory responses, what contributes to more severity of the disease [30, 31].

The participants of this study did not show values of serum levels of CRP that were indicative of the presence of inflammation. Differently, Yang et al. [32] in a study of retrospective cohort with hospitalized patients observed that patients with COVID-19 presented increased levels of CRP, which is related to the severity of the disease. It is believed that patients with increased levels of CRP in severe viral infections, such as COVID-19, are five times more likely to develop SARS [33].

In this context, CRP may have application as a sensitive serum marker of inflammation and severe infection in COVID-19. When cells are infected by SARS-CoV-2, they recruit macrophages, causing an increase in the vascular permeability as an acute-phase disease response. From then on, the death of infected cells occurs causing tissue damage, which, in turn, can lead to tissue hypoxia, and the triggering of the immune system response contributes to the activation of a chain of proinflammatory cytokines, a phenomenon known as a cytokine storm [34].

The exacerbated production of the markers of lipid peroxidation and inflammation is a normal characteristic of the physiology of COVID-19; thus, an adequate intake of vitamin E must be assured to patients with COVID-19 with the aim of preventing nutritional deficiencies and then, contribute to boost the immune response, the antioxidant system and the fight against inflammations by means of the biological role of this vitamin.

The present study main limitations are: (1) a cross-sectional outlining limiting the inference of the results; (2) the nondetermination of the plasma concentration of vitamin E, what could bring complementary information to the dietary intake and to the nutritional status related to this vitamin and to allow greater statistical power in the tests with the lipid peroxidation markers; (3) the reliability in the dietary intake evaluation, due to the fact it depends on several factors, such as memory, what could underestimate this analysis, even with the corrections of inter- and intrapersonal variability being performed; (4) more specific inflammatory markers, such as IL-6, were not determined, which were not analyzed due to operational difficulties.

However, this research can be considered as novel since it was conducted with patients assisted in BHUs with mild symptoms and still in the beginning of the disease, considering the benchmark of the moment of the diagnosis with the use of gold-standard tests for the disease diagnosis. In addition, there is no sufficient evidence regarding the coronavirus disease, dietary intake, and oxidative stress markers. Given the long duration of the pandemic, the population had a significant use of vitamin and mineral supplements, with or without medical prescription, that had no evidence supporting their efficacy. Besides, the study was performed in a timeframe when there were still no vaccines and drugs with proven efficacy for the treatment of the disease, being interrupted when new variants and vaccines began to be provided to the Brazilian population in general to prevent bias, and thus limiting the sample size.

## 5. Conclusion

The dietary intake of vitamin E was weakly correlated with lipid peroxidation as determined by the content of MDA. In addition, there was a moderate correlation between the ingestion of this vitamin and monounsaturated fats, as well as a weak correlation with the ingestion of polyunsaturated fats. No significant correlation was found between serum concentrations of CRP and vitamin E dietary intake.

This study may be the starting point for new research on this novel subject. The assessment of the specific nutritional recommendations would be of paramount importance for prevention, care, and treatment of microbial-based diseases.

## Figures and Tables

**Table 1:** Social-economical characteristics of patients with flu symptoms assisted by basic health units and with a positive diagnosis for COVID-19 through RT-PCR; Teresina, Piauí, 2021.

Variables	Positive COVID-19 (*n* = 121)	
	No.	%
Sex		
Male	66	54.5
Female	55	45.5
Age (y/o)	Mean (SD)	Md (min–max)
	34.22 (10.4)	33 (18.0–57.0)
Income (MW)		
No income	18	14.9
Welfare	3	2.5
1SM	51	42.1
>1SM	49	40.5
Schooling		
Illiterate	1	0.8
Literate	9	7.4
Complete elementary school	20	16.5
Complete high school	64	52.9
Complete college degree	27	22.3
Previous diagnosis of NCDs		
Yes	24	19.8
No	97	80.2
Use of medication		
Yes	22	18.2
No	99	81.8
Physical activity		
Yes	50	41.3
No	71	58.7

*Note*. MW = minimum wage; Md = median; NCDs = noncommunicable diseases.

**Table 2:** Clinical characteristics of patients with symptoms of flu syndrome assisted by basic health units and with a positive diagnosis for COVID-19 through RT-PCR; Teresina, Piauí, 2021.

Variables	Positive COVID-19 (*n* = 121)	
	No.	%
Days with symptoms		
3 days	35	28.9
4 days	31	25.6
5 days	32	26.4
6 days	11	9.1
7 days	12	9.9
Fever		
Yes	76	62.8
No	45	37.2
Cough		
Yes	74	61.2
No	47	38.8
Shortness of breath		
Yes	23	19.0
No	98	81.0
Loss of smell		
Yes	52	43.0
No	69	57.0
Loss of taste		
Yes	45	37.2
No	76	62.8
Headache		
Yes	83	68.6
No	38	31.4
Body pain		
Yes	75	62.0
No	46	38.0
Diarrhea		
Yes	21	17.4
No	100	82.6
Nausea		
Yes	17	14.0
No	104	86.0
Vomiting		
Yes	3	2.5
No	118	97.5
Sore throat		
Yes	16	13.2
No	105	86.8
Coryza		
Yes	22	18.9
No	99	81.8
Nasal congestion		
Yes	12	9.9
No	109	90.1
Abdominal pain		
Yes	16	13.2
No	105	86.8
Ocular pain		
Yes	3	2.5
No	118	97.5

**Table 3:** Mean values and standard deviations of anthropometric parameters of patients with symptoms of flu syndrome assisted in basic health units with positive diagnosis for COVID-19 through RT-PCR; Teresina, Piauí, 2021.

Variables	Mean ± SD (*n* = 121)	*N* (%)	*p* ^∗^
Body weight (kg)	75.30 ± 17.53		
Height (m)	1.66 ± 0.98		
BMI (kg/m^2^)	26.91 ± 5.88		
WC (cm)	89.22 ± 14.78		
Male			
No risk		37 (56.1)	0.008
High risk		16 (24.2)	
Very high risk		13 (19.7)	
Female			
No risk		19 (34.5)	
High risk		11 (20.0)	
Very high risk		25 (45.5)	

*Note*. ^∗^Chi-square test; SD = standard deviation; BMI = body mass index; WC = waist circumference.

**Table 4:** Means values and standard deviations of energy, macronutrients, and vitamin E of patients with symptoms of flu syndrome assisted by the basic health units with positive diagnosis for COVID-19 through RT-PCR; Teresina, Piauí, 2021.

Variables	Mean ± SD (*n* = 121)	Adequation (%)
Energy (kcal/day)	1,365.22 ± 315.85	–
Carbohydrates (g/day)	183.44 ± 52.02	77.6
Proteins (g/day)	71.09 ± 17.56	100
Lipids (g/day)	42.52 ± 11.7	96.7
Polyunsaturated fats (g/day)	13.74 ± 3.62	–
Monounsaturated fats (g/day)	5.82 ± 1.13	–
Saturated fats (g/day)	10.95 ± 1.91	–
Vitamin E (mg/day)	3.25 ± 1.64	–

*Note*. Carbohydrates = 45%–65%; proteins = 10%–35%; lipids = 20%–35%; Adequation (%) = 90%–110%; EAR: Vitamin E = 15 mg/day [13].

**Table 5:** Values of plasma concentrations of MDA, MPO, and CRP in patients with flu-like symptoms treated at basic health units with a positive diagnosis of COVID-19 by RT-PCR; Teresina, Piauí, 2021.

Variables	Mean ± SD (*n* = 121)
MDA (nmol/mL)	4.23 ± 1.01
MPO (U/*µ*L)^a^	2.78 (1.39 − 4.17)
CRP^a^	4.86 (2.77 − 10.29)

*Note*. ^a^Values presented as mean and interquartile interval; MDA = malondialdehyde; MPO = myeloperoxidase; CRP = C-reactive protein.

**Table 6:** Coefficients of correlation between the intake of vitamin E, lipids, saturated, monounsaturated and polyunsaturated fats, body mass index, malondialdehyde, and myeloperoxidase of patients with symptoms of flu syndrome assisted by the basic health units with a positive diagnosis for COVID-19 through RT-PCR; Teresina, Piauí, 2021.

	Lipids	Sat_F	Mono_F	Poly#_F	BMI	MDA	MPO#	PCR
Vitamin E	*r* = 0.337	*r* = 0.299	*r* = 0.483	*r* = 0.269	*r* = 0.029	*r* = 0.197	*r* = 0.083	*r* = −0.131
	*p* ≤ 0.001	*p*=0.001	*p* ≤ 0.001	*p*=0.003	*p*=0.751	*p*=0.031	*p*=0.368	*p*=0.153

*Note*. Pearson's correlation (*p* < 0.05) # Sperman's correlation; Vit E = vitamin E; Sat_F = saturated fats; Mono_F = monounsaturated fats; Poly_F = polyunsaturated fats; BMI = body mass index; MDA = malondialdehyde; MPO = myeloperoxidase.

## Data Availability

Data will be available under request.
